# Zoledronic Acid-Loaded β-TCP Inhibits Tumor Proliferation and Osteoclast Activation: Development of a Functional Bone Substitute for an Efficient Osteosarcoma Treatment

**DOI:** 10.3390/ijms22041889

**Published:** 2021-02-14

**Authors:** Yuka Kameda, Mamoru Aizawa, Taira Sato, Michiyo Honda

**Affiliations:** 1 Department of Applied Chemistry, School of Science and Technology, Meiji University, 1-1-1 Higashimita, Tama-ku, Kawasaki 214-8571, Kanagawa, Japan; mary.1256@icloud.com (Y.K.); mamorua@meiji.ac.jp (M.A.); 2 Organization for the Strategic Coordination of Research and Intellectual Properties, Meiji University, 1-1-1 Higashimita, Tama-ku, Kawasaki 214-8571, Kanagawa, Japan; tsato@college.fdcnet.ac.jp

**Keywords:** Zoledronic acid, β-TCP, osteosarcoma, bioresorbability

## Abstract

Osteosarcoma has a poor survival rate due to relapse and metastasis. Zoledronic acid (ZOL), an anti-resorptive and anti-tumor agent, is used for treating osteosarcoma. Delivery of ZOL to the target region is difficult due to its high binding affinity to bone minerals. This study developed a novel treatment for osteosarcoma by delivering ZOL to the target region locally and sustainably. In this study, we fabricated a novel bone substitute by loading ZOL on β-tricalcium phosphate (β-TCP). The ZOL-loaded β-TCP (ZOL/β-TCP) would be expected to express the inhibitory effects via both bound-ZOL (bound to β-TCP) and free-ZOL (release from ZOL/β-TCP). To explore the ability to release ZOL from the ZOL/β-TCP, the amount of released ZOL was measured. The released profile indicates that a small amount of ZOL was released, and most of it remained on the β-TCP. Our data showed that ZOL/β-TCP could successfully express the effects of ZOL via both bound-ZOL and free-ZOL. In addition, we examined the biological effects of bound/free-ZOL using osteosarcoma and osteoclasts (target cells). The results showed that two states of ZOL (bound/free) inhibit target cell activities. As a result, ZOL/β-TCP is a promising candidate for application as a novel bone substitute.

## 1. Introduction

Osteosarcoma is a well-known malignant bone tumor that surfaces in childhood and adolescence. Moreover, osteosarcoma exhibits highly aggressive and early systemic metastasis [1]. Over 90% of patients with osteosarcoma die of pulmonary metastasis [2]. Therefore, novel treatments for osteosarcoma need to be developed to improve the outcome. 

The bone microenvironment acts as a fertile ground for the growth of cancer cells [3,4]. Bone tissues contain various growth factors, including transforming growth factor beta (TGF-β), insulin-like growth factor (IGF), fibroblast growth factor (FGF), platelet-derived growth factor (PDGF), and bone morphogenetic proteins (BMPs). These growth factors can both directly and indirectly promote the growth of tumor cells. Tumor-derived factors such as parathyroid hormone-related protein (PTH-rP) also promote the secretion of the receptor activator of nuclear factor kappa-B ligand (RANKL) by osteoblast lineage cells, such as osteoblast precursors, osteoblasts, and osteocytes. In addition, interleukin-6 (IL-6), interleukin-11 (IL-11), and tumor necrosis factor alpha (TNF-α) produced in tumor cells also enhance osteoclast formation and activation or osteoclast precursor activation. The secretion of growth factors in bone stimulates the growth of metastases by abnormal turnover. Tumor cell progression is related to the abnormal activation of osteoclasts.

Currently, surgery, radiotherapy, and chemotherapy are being used for the treatment of osteosarcomas [5,6]. However, these treatments cannot remove the tumor cells and suppress osteoclast activation. As previously reported, about 11% of patients with osteosarcoma survive after treatment with a combination of surgery and chemotherapy [7]. Moreover, the survival of patients with relapse is reported to be less than 30% [8]. Relapse and metastasis are the barriers to the successful treatment of osteosarcoma. 

Bisphosphonates (BPs) are a class of drugs that prevent the loss of bone density. They are widely used for treating osteoporosis and other malignant bone metastases. Among BPs, nitrogen-containing BPs (N-BPs), which are anti-resorptive medications, inhibit farnesyl pyrophosphate synthase (FPPS) activity in the mevalonate pathway [9,10,11]. BPs contain two phosphonate groups that share a carbon atom (P-C-P). The common backbone structure of P-C-P has a binding affinity for bone minerals, such as calcium phosphate (CaP). The R2 chain determines the anti-resorptive properties of BP (Figure S1). The R1 structure is commonly a hydroxyl group. The anti-resorptive abilities of N-BPs (e.g., alendronate and pamidronate) are up to 100 times more potent than non-nitrogen-containing BPs, such as clodronate and etidronate [12]. Furthermore, N-BPs that contain amino groups or heterocyclic rings (e.g., risedronate and zoledronic acid) are the most potent anti-resorptive agents among BPs [12]. N-BPs have an anti-tumor effect on breast and prostate cancers [13,14].

N-BPs are administered orally or intravenously. However, N-BPs bind to bone minerals due to their high binding affinity. The remaining N-BPs are rapidly removed from internal circulation [15]. N-BPs are used to prevent metastasis from other host tissues [16,17] However, the disadvantage of N-BPs is that they are difficult to deliver to the target region. Therefore, developing novel methods for delivering N-BPs to the target region is a present need. 

To develop an effective treatment for osteosarcoma, we fabricated a novel bioresorbable bone substitute that consists of β-tricalcium phosphate (β-TCP) loaded with zoledronic acid (ZOL). ZOL, containing an imidazole ring as the R2 chain (Figure S1), has excellent anti-resorptive and anti-tumor effects through inhibition of osteoclast activity and bone resorption [12,18]. ZOL loaded on β-TCP (ZOL/β-TCP) would work in two states: (i) binding to β-TCP (bound-ZOL), and (ii) release ZOL from ZOL/β-TCP (free-ZOL). The release of ZOL is sustained by both Ca^2+^ dissolution from β-TCP and osteoclast-mediated resorption. Recently, CaP powders, cement, and coatings have been proposed as alternatives to the local administration of N-BPs [19,20]. The functionalization of hydroxyapatite (HAp) and octa calcium phosphate (OCP) with N-BPs has been reported [21,22,23]. OCP can be easily converted into HAp because of its high dissolubility and its structural similarity to that of HAp [23]. When functionalized with N-BPs, OCP rapidly releases N-BPs with Ca^2+^ dissolution. However, N-BPs are not released from HAp loaded with N-BPs because of the low dissolubility of Ca^2+^ and a nonbioabsorbable property [24]. To achieve local and sustained release of N-BPs, we focused on β-TCP, which has a dissolubility between those of HAp and OCP [24].

The aim of the present study is to develop a carrier that releases ZOL locally and sustainably. In this study, we fabricated ZOL/β-TCP and examined the material and biological properties of ZOL/β-TCP. Finally, we demonstrated its usefulness in the local and sustainable treatment of osteosarcoma in vitro. 

## 2. Results

### 2.1. Characterization of the ZOL-Loaded β-TCP

#### 2.1.1. Adsorption of ZOL on β-TCP Powders

To prepare the ZOL/β-TCP powders, β-TCP powders were immersed in ZOL solutions of different concentrations. The adsorption of ZOL onto β-TCP powders, which was dose-dependent, increased as the concentrations of ZOL solutions were increased (Table 1). As previously reported, BP can bind or chelate with Ca^2+^ [25,26]. Theoretically, the interaction between CaP and BP is reversible. The profiles of the BP interactions with CaP displayed Langmuir adsorption isotherms: in other words, an equilibrium between adsorbate and adsorbent system. However, the adsorption of ZOL onto the β-TCP powders did not reach a plateau until the concentration of ZOL solution was 12 mmol/L (Figure S2), and the saturated adsorption amount of ZOL was unclear. These data showed that the binding capacity of ZOL to β-TCP was sufficient, and a greater amount of ZOL could bind to β-TCP. 

#### 2.1.2. Zeta Potential

Zeta potential indicates the change in surface charge on the CaP particles and the binding affinity of BPs to CaP surfaces [21,27]. The zeta potentials of ZOL/β-TCP powders at pH 7.3 and 5.5 were examined (Table 1). As a result, the zeta potential of the ZOL(0)/β-TCP powders were negatively charged (−9.46 ± 1.76 mV at pH 7.3 and −12.25 ± 2.41 mV at pH 5.5). When the ZOL was loaded onto the β-TCP powders, the zeta potential started to decrease sharply at pH 7.3. In contrast, the zeta potential at pH 5.5 shifted less sharply. A previous study reported that this change in the zeta potential is due to the charges on the phosphonate and R2 functional groups [27]. Therefore, the zeta potentials indicated that the binding states of ZOL to β-TCP were changed by the surrounding pH.

#### 2.1.3. X-ray Diffractometry (XRD) Patterns

**Figure 1:**
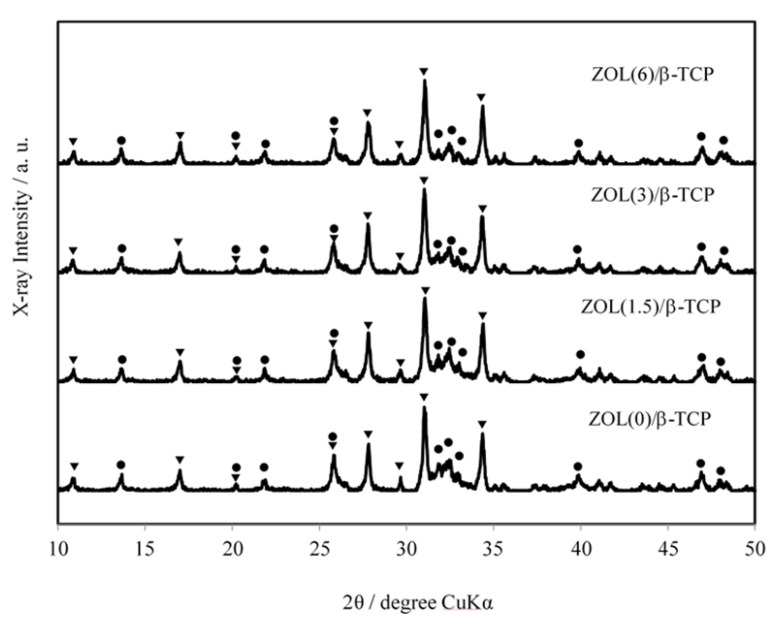
XRD patterns of zoledronic acid (ZOL)/β-tricalcium phosphate (β-TCP) powders. XRD patterns of the ZOL/β-TCP powders. ▼; β-TCP, ●; hydroxyapatite (HAp).

To identify the crystalline phase of the ZOL/β-TCP powders, we explored the XRD patterns of the ZOL/β-TCP powders. The results are illustrated in Figure 1. The ZOL/β-TCP powders consisted mainly of two crystalline phases: β-TCP and HAp. During manufacturing, a small amount of HAp with low crystallinity was formed through the hydration of β-TCP. In addition, the conversion of HAp decreased with an increase in the amount of loaded ZOL. At 10 day after preparation, the conversion rate was 55% for ZOL(0)/β-TCP, 48% for ZOL(1.5)/β-TCP, 30% for ZOL(3)/β-TCP, and 23% for ZOL(6)/β-TCP, respectively. Therefore, loading the ZOL on β-TCP powders will affect the HAp growth of β-TCP.

#### 2.1.4. Solubility of Ca^2+^ from ZOL/β-TCP Powders

**Figure 2:**
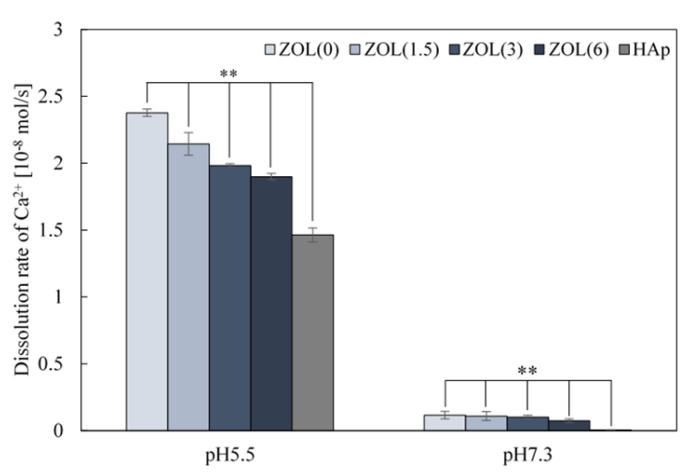
Dissolution of Ca^2+^ of the ZOL/β-TCP powders. Dissolution rate of Ca^2+^ of the ZOL/β-TCP powders in Acetate buffer (pH 5.5) and Tris-HCl buffer (pH 7.3). Error bars indicate standard deviation of the mean. The asterisks show ** *p* < 0.01 as compared with the dissolution rate of HAp by Student’s *t*-test.

The strong binding affinity of BPs—including ZOL—to CaP, prevents the growth and dissolution of the latter [27,28]. To assess the binding affinity of ZOL to the β-TCP powders, the Ca^2+^ dissolution rate of the ZOL/β-TCP powders was measured at pH 7.3 (physiological condition) and pH 5.5 (osteoclast activation), in accordance with JIST0330-3 (Figure 2). At pH 5.5, the dissolution rate decreased as the amount of ZOL increased. However, the dissolution rate of each of the ZOL/β-TCP powder was higher than that of the HAp powder. At pH 7.3, the dissolution rate of ZOL/β-TCP powders slightly decreased as the amount of loaded ZOL was increased. The results indicated that the ZOL/β-TCP powders have higher Ca^2+^ dissolubility than HAp, even though ZOL inhibited Ca^2+^ dissolution.

#### 2.1.5. Release of ZOL from ZOL/β-TCP

**Figure 3:**
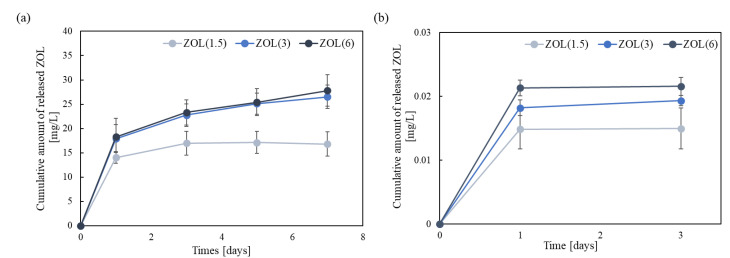
Cumulative amount of released ZOL from ZOL/β-TCP. Cumulative amount of released ZOL from (**a**) ZOL/β-TCP powders and (**b**) ZOL/β-TCP disc in Tris-HCl buffer (pH 7.3). Error bars indicate standard deviation of the mean.

To identify the ability to release ZOL from the ZOL/β-TCP powder, we examined the cumulative amount of released ZOL (Figure 3a). The amount of released ZOL was low at pH 7.3. The amount and behavior of ZOL release from ZOL(3, 6)/β-TCP powders was almost unchanged. Moreover, the rate of release of ZOL decreased with an increase in the amount of loaded ZOL (data not shown). Therefore, it was confirmed that ZOL released in very limited amounts from ZOL/β-TCP powders, due to its high binding affinity to CaP. 

Next, we examined the amount of ZOL released from the ZOL/β-TCP disc to identify the ability to release ZOL (Figure 3b). The ZOL/β-TCP disc released ZOL depending on the amount of loading ZOL at pH 7.3, although the observed difference was not significant. In addition, the amount of released ZOL did not change from 24 h to 72 h. The amount of ZOL released from ZOL/β-TCP discs inhibited the proliferation and activation of tumor cells and osteoclasts [29,30]. We expected that the ZOL released from the ZOL/β-TCP disc would have inhibitory effects as free-ZOL. In this study, we did not measure the amount of released ZOL in the culture medium because the concentrations of various ions, such as Ca^2+^ and PO_4_^3−^, in the culture medium were different. Therefore, the actual process of released ZOL will be different. In this examination, we confirmed that the ZOL/β-TCP disc released ZOL at pH 7.3, and the data showed that the free-ZOL existed in the elution medium (EM). 

The amount of ZOL exposed on the surface of the ZOL/β-TCP disc was about 4 mass% of the amount of ZOL actually loaded. The rates of released ZOL were low (ZOL(1.5)/β-TCP disc; 1.36 ± 0.244 mass%; ZOL(3)/β-TCP disc; 0.837 ± 0.0502 mass%; ZOL(6)/β-TCP disc; 0.490 ± 0.0411 mass%). Therefore, the data indicated that the ZOL remained on the surface of each ZOL/β-TCP disc after immersion in the culture medium for 24 h. 

### 2.2. Effects of ZOL/β-TCP on Osteoblasts and Tumor Cells

Two states of ZOL, bound-ZOL and free-ZOL, would work on the target cells (tumor cells and osteoclasts). We first determined the loading concentration of ZOL, which inhibits tumor cell proliferation but not osteoblast proliferation. In this study, HOS and MC3T3-E1 cells were cultured on ZOL/β-TCP discs or in the EM formed from ZOL(X)/β-TCP discs (EM(X)). Prior to the test, we confirmed that ZOL was not released from the ZOL/β-TCP disc after 24 h of immersion. To examine the effect of bound-ZOL, we used the immersed ZOL/β-TCP disc that did not contain free-ZOL. EM, containing only free-ZOL, was obtained from a culture medium where ZOL/β-TCP discs were immersed for 24 h. 

#### 2.2.1. Anti-Tumor Effects

**Figure 4:**
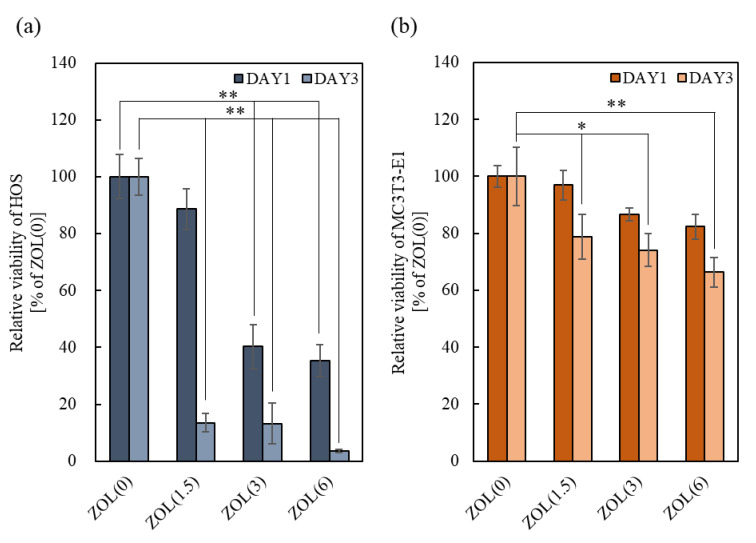
Effect of bound-ZOL on cell viability of tumor cells and osteoblasts. HOS and MC3T3-E1were cultured on each ZOL/β-TCP disc for three days. After culturing, the relative viability of (**a**) HOS and (**b**) MC3T3-E1 was determined by MTT assay. Error bars indicate standard deviation of the mean. The asterisks show * *p* < 0.05 and ** *p* < 0.01 as compared with ZOL(0) by Student’s *t*-test.

**Figure 5:**
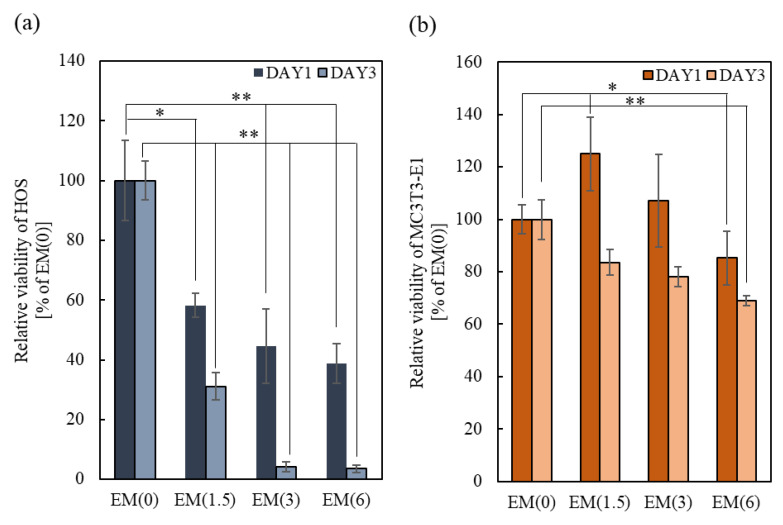
Effect of free-ZOL on cell viability of tumor cells and osteoblasts. HOS and MC3T3-E1were cultured in each elution medium of ZOL/β-TCP for 3 days. After culturing, relative viability of (**a**) HOS and (**b**) MC3T3-E1 were determined by 3-(4, 5-Dimethylthiazol-2-yl)-2,5-diphenyltetrazoloum bromide (MTT) assay. Error bars indicate standard deviation of the mean. The asterisks show * *p* < 0.05 and ** *p* < 0.01 as compared with EM(0) by Student’s *t*-test.

Owing to the effects of bound-ZOL on tumor cell proliferation, the relative viability of HOS significantly decreased after 3 days of culture with increasing amounts of loaded ZOL (Figure 4a). Morphological observations showed that HOS adhered to the ZOL(0, 1.5)/β-TCP discs (Figure S3a). However, unlike the cells cultured on ZOL(0)/β-TCP disc, HOS expanded or contracted on the ZOL(1.5)/β-TCP disc. Moreover, we observed the apoptotic-like morphology of ZOL(1.5)/β-TCP. HOS cells were not found on the ZOL(3, 6)/β-TCP discs. In contrast, the viability of HOS cultured in EM also decreased, and the inhibition of cell proliferation time-dependently accelerated, similar to the effect of bound-ZOL (Figure 5a). Unlike the normal morphology of HOS (in EM(0)), the morphology of HOS in EM(1.5, 3) expanded or contracted, which was similar to the effect of bound-ZOL (Figure S4a). These data demonstrated that ZOL(1.5, 3, 6)/β-TCP discs exhibited anti-tumor effects, to which bound-ZOL and free-ZOL contributed. 

#### 2.2.2. Cell Viability of Osteoblasts

The effect of bound-ZOL on osteoblast viability is shown in Figure 4b. After 3 days of culture, the cell viability of MC3T3-E1 cultured on ZOL(6)/β-TCP discs significantly decreased. However, no obvious differences among all samples were seen on day 1. It shows that the slightly released ZOL and ions can affect cell survival. As for cell morphology, no abnormality of MC3T3-E1 was observed (Figure S3b). To evaluate the effects of free-ZOL on MC3T3-E1 cells, the cells were cultured in EM (Figure 5b). The relative viability of MC3T3-E1 cells was reduced by 16.4% for EM(1.5), 21.9% for EM(3), and 31.3% for EM(6). However, the cell morphology did not change (Figure S4b). These data demonstrate that a high dose of ZOL (ZOL(6)/β-TCP) affects the cell viability of MC3T3-E1 via bound/free-ZOL.

### 2.3. Effects of ZOL/β-TCP on Osteoclast Differentiation and Activation

**Figure 6:**
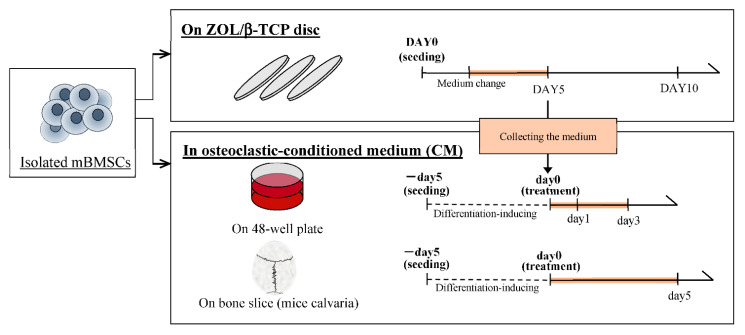
Schedule of examination of mBMSCs differentiation and activation.

To examine the effects of bound-ZOL and free-ZOL on osteoclast formation and activation, mice bone marrow stromal cells (mBMSCs) were introduced to differentiate into osteoclasts (Figure 6). Cells were cultured on ZOL/β-TCP discs or in an osteoclastic (on ZOL(X)/β-TCP disc) conditioned medium (CM(X)). Released ZOL was not detected in the medium when ZOL/β-TCP discs were immersed for 24 h. Therefore, CM included ZOL only released by osteoclast-mediated resorption of ZOL/β-TCP. In this examination, both bound and free-ZOL are expected to express inhibitory effects on osteoclasts when cultured on ZOL/β-TCP discs.

#### 2.3.1. Inhibitory Effects for Osteoclast Formation and Activation on ZOL/β-TCP Disc

To examine the effects of ZOL/β-TCP on osteoclast proliferation and differentiation, mBMSCs were cultured on each ZOL/β-TCP disc. No adherent cells were seen on the ZOL(6)/β-TCP disc after 3 days of culturing (Figure S5). These results revealed that mBMSCs did not differentiate into osteoclasts on ZOL(6)/β-TCP discs. Therefore, we examined the effects of ZOL(0, 1.5, 3)/β-TCP on osteoclasts, through the following experiments. 

**Figure 7:**
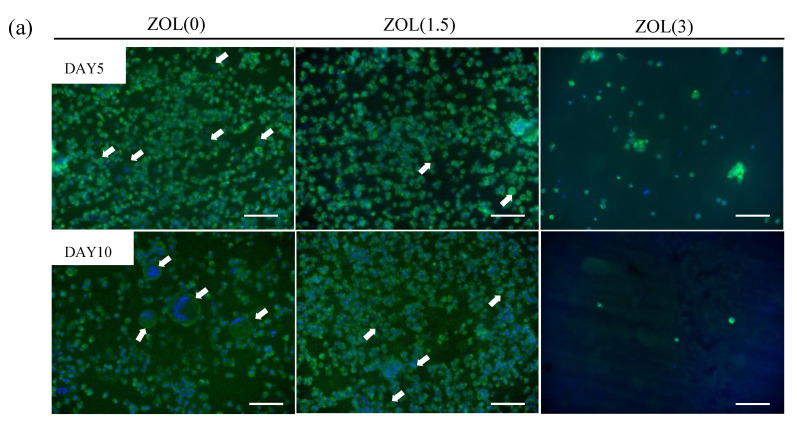

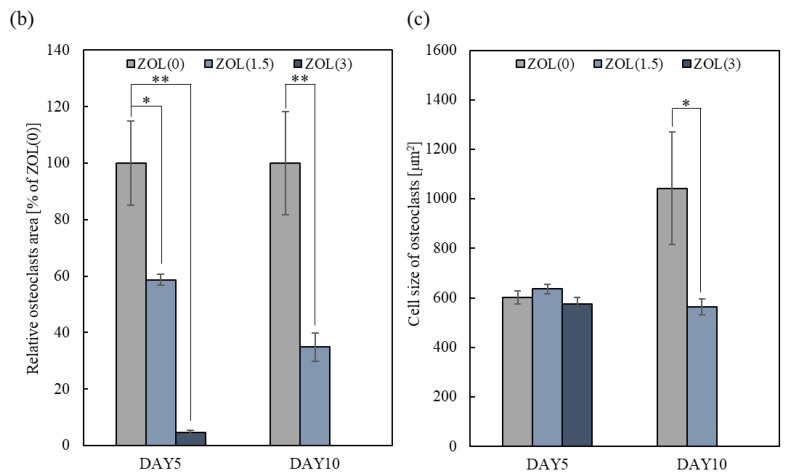
Effect of bound-ZOL for mice bone marrow stromal cells (mBMSCs) differentiation. mBMSCs were cultured on each ZOL/β-TCP disc for 5, 10 days (DAY5, 10). (**a**) Morphological observation of mBMSCs at days 5 and 10. mBMSCs were stained with Alexa Flour^®^ 488-labeled phalloidin for F-actin (green) and 4’,6-diamidino-2-phenylindole (DAPI) for nuclei (blue) and observed with fluorescence microscopy. White arrows; osteoclasts (cell with more than 2 nuclei), scale bars; 100 μm. (**b**) Relative osteoclasts area and (**c**) cell size of osteoclasts were analyzed by image analyzer. Error bars indicate standard deviation of the mean. The asterisks show * *p* < 0.05, ** *p* < 0.01 as compared with ZOL(0) by Student’s *t*-test.

**Figure 8:**
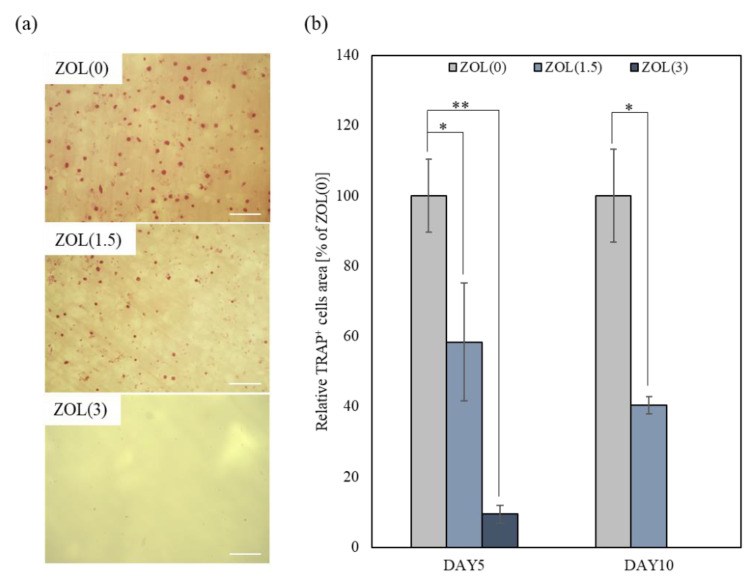
Effect of bound-ZOL for osteoclast activation. mBMSCs were cultured on each ZOL/β-TCP disc for 5 and 10 days (DAY5, DAY10). (**a**) Observation images of TRAP activity staining at day 10. Red; TRAP positive (TRAP^+^) cells, scale bars; 200 μm. (**b**) Relative TRAP^+^ cells area was analyzed by image analyzer. Error bars indicate standard deviation of the mean. The asterisks show * *p* < 0.05 and ** *p* < 0.01 as compared with ZOL(0) by Student’s *t*-test.

A previous study reported that mBMSCs became adherent and differentiated after 3 days of culturing and formed fully mature multinucleated osteoclasts after 5 days of culturing [31]. In this study, we cultured mBMSCs on ZOL/β-TCP discs for 5 and 10 days and examined osteoclast formation and activation. Multinucleate cells were observed on the ZOL(0, 1.5)/β-TCP discs after 5 and 10 days of culturing (Figure 7a). However, no adherent cells or multinucleate cells were seen on the ZOL(3)/β-TCP disc. Based on the above results, we analyzed two indexes: osteoclast area (more than 2 nuclei) and osteoclast cell size (Figure 7b,c). After the culture period, the size of the osteoclast area decreased as the amount of loaded ZOL was increased. The cell sizes of the osteoclasts were either unchanged or decreased, even though osteoclasts grew up on the ZOL(0)/β-TCP disc. Additionally, to examine the osteoclast activation on the ZOL/β-TCP discs, tartrate-resistant acid phosphatase (TRAP)-staining was performed (Figure 8). TRAP-positive (TRAP^+^) cells were observed on the ZOL(0, 1.5)/β-TCP discs after 10 days of culture, although the number of cells decreased with an increase in the amount of loaded ZOL. The pit area formed by osteoclast resorption was also observed on the ZOL(0)/β-TCP disc after 10 days of culturing (Figure S6).

Osteoclasts were particularly activated on ZOL(0, 1.5)/β-TCP discs (Figure 6 and Figure 7). ZOL was released from ZOL(1.5)/β-TCP disc by osteoclast-mediated resorption. These data showed that both bound-ZOL and free-ZOL inhibited osteoclast formation and differentiation on the ZOL(1.5)/β-TCP disc. ZOL would also be released from the ZOL(3)/β-TCP disc. mBMSCs on ZOL(3)/β-TCP discs did not differentiate into osteoclasts, and osteoclast-mediated resorption also did not occur. Osteoclast proliferation and activation were inhibited by contact with the surface of the discs. However, it remains unclear how the inhibitory effects are expressed on bound-ZOL and free-ZOL. 

#### 2.3.2. Effects of Free-ZOL on Osteoclast Activation

To evaluate the effects of free-ZOL of ZOL/β-TCP on osteoclasts, mBMSCs were cultured in a differentiation-induced medium for 5 days on 48-well plates. The differentiation and activation of osteoclasts were confirmed (data not shown). As shown in Figure 8a and Figure S7, multinucleated cells and their filopodia were observed in CM(0). A previous study revealed that activated osteoclasts had filopodia [32]. In contrast, after treatment for 1 day, we observed an abnormal morphology in CM(3), which is an advanced apoptosis of osteoclasts, similar to the previously reported morphology [33]. 

**Figure 9:**
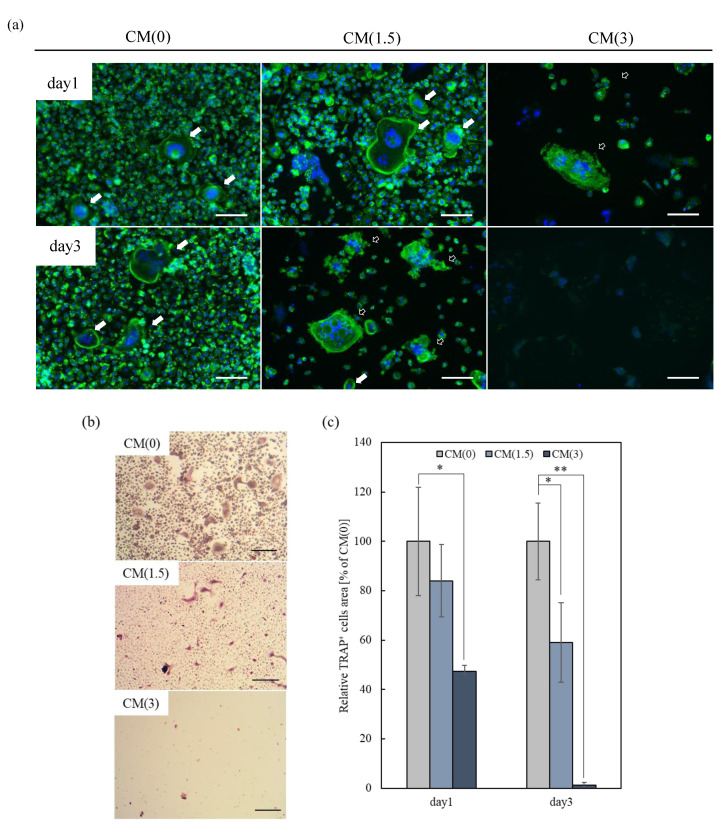
Effect of free-ZOL for mBMSCs activation. mBMSCs were treated with osteoclastic (on the ZOL/β-TCP disc) conditioned medium for 1, 3 days (day 1, 3). (**a**) Morphological observation of mBMSCs. mBMSCs were stained with Alexa Flour^®^ 488-labeled phalloidin for F-actin (green) and DAPI for nuclei (blue) and observed by fluorescence microscopy. Scale bars; 100 μm, filled white arrows; osteoclasts (cell with more than 2 nuclei), open white arrows; abnormal shape of osteoclasts. (**b**) Observation images of TRAP activity staining at day 3. Red; TRAP^+^ cells, scale bars; 200 μm. (**c**) Relative TRAP^+^ cells area was analyzed by image analyzer. Error bars indicate standard deviation of the mean. The asterisks show * *p* < 0.05, ** *p* < 0.01 as compared with conditioned medium 0 (CM(0)) by Student’s *t*-test.

A similar abnormal morphology was also observed in CM(1.5) after treatment for 3 days. Moreover, the number of TRAP^+^ cells in CM(1.5, 3) also decreased (Figure 9b,c). 

**Figure 10:**
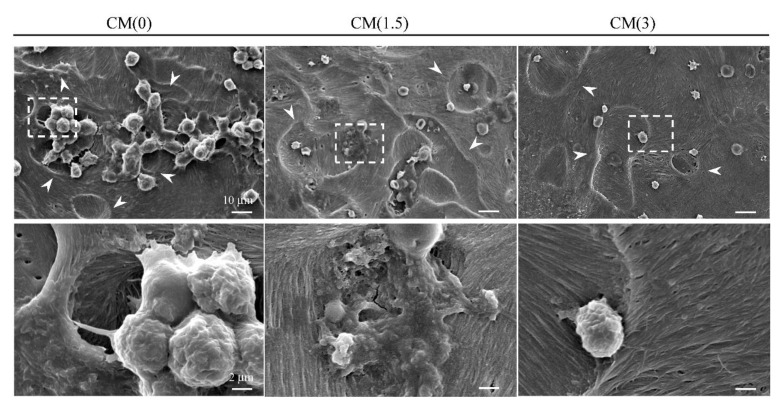
In vitro osteoclast-mediated bone resorption assay. mBMSCs were treated with osteoclastic (on the ZOL/β-TCP disc) conditioned medium on bone slice (mice calvaria) for 5 days. The surface of the bone slice was observed with SEM. The bottom panel shows magnification images of the dotted rectangle boxes in upper panel. Scale bars; 10 μm (upper panel), 2 μm (bottom panel), pit area; white arrow heads.

Next, to examine the effects of free-ZOL on osteoclast activation around bone tissues, we designed an in vitro osteoclast-mediated bone resorption assay using bone slices of a mouse’s calvaria region. The assay is structured in the form of a protocol (Figure 6). mBMSCs were differentiated into osteoclasts on bone slices after treatment with CM for 5 days. As shown in Figure 10, we successfully differentiated mBMSCs into osteoclasts on the bone slices under normal culture conditions (in a differentiation-induced medium with data not shown). In CM(0), the morphology of the filopodium and resorption area was observed (Figure 10). Therefore, we concluded that osteoclasts maintained their activation in CM(0). However, we did not observe an osteoclast-like morphology (filopodium), although a large resorption area was observed. These results imply that treatment with CM(1.5, 3) inhibited the resorption of bone slices by activated osteoclasts. Taken together, both β-TCP dissolution and osteoclast-mediated resorption could supply ZOL.

## 3. Discussion

The current treatment of bone diseases such as osteosarcoma did not prevent relapse from the remaining tumor cells and activation of osteoclasts. For treating osteosarcoma, chemotherapeutic agents are widely used. N-BPs have also been administered to treat malignant bone disease [34], although N-BPs rapidly bind to bone minerals and are difficult in terms of getting delivered to the target region during treatment. Therefore, the development of novel methods that can not only prevent relapse but also locally and effectively deliver N-BPs is necessary. 

In this study, we prepared ZOL/β-TCP to treat osteosarcoma. In particular, it is assumed that ZOL/β-TCP will be applied to bone cement. Currently, in the surgical treatment of osteosarcoma, the tumor is removed by curettage, and the bone substitute is filled. ZOL/β-TCP is a novel material with functionality, which is in lieu of existing bone substitutes. As a drug delivery system, the injection of calcium phosphate cement has enticed widespread attention recently, and diverse formulations have been proposed [35]. Bone cement using β-TCP or BPs has been reported [36,37]. The ZOL/β-TCP resolves the problem of not being able to supply the target region and can therefore administer BPs. In addition, it was reported that N-BP administration in oral or intravenous had various side effects. Therefore, the ZOL/β-TCP enables the delivery of ZOL to the target region to prevent their invasion [38,39,40]. We expected that ZOL/β-TCP could be applied to bone cement, and examined the ZOL/β-TCP effects for target cells using ZOL/β-TCP in an in vitro study. We provide new insights for the development of functional bone cement in this study.

ZOL strongly binds to CaP and has strong anti-resorptive abilities [27]. The amount of ZOL loaded onto β-TCP did not reach a plateau (Table 1 and Figure S2). These data imply that specific surface areas of the β-TCP powders were sufficient to bind ZOL. Moreover, it interacts with β-TCP and ZOL via both chelating and other binding states (i.e., electrostatic interaction). Therefore, the binding capacity of β-TCP with ZOL can expand through various binding states.

The possible structural interactions of BPs with CaP, including bone minerals, has been investigated [21,22,25,27]. The positively charged N-BPs (alendronate, ibandronate, and ZOL) can create a positive CaP surface to attract negatively charged phosphate moieties [25]. The induction of phosphate moieties, therefore, will augment the accumulation of ZOL and increase the maximum binding capacity of β-TCP. Thus, when ZOL binds to CaP, the surface charge of CaP can influence the subsequent binding of charged molecules, for example, the ability to attract other ions or charged molecules [27]. Moreover, ZOL will also form electrostatic interactions or intramolecular connections with the hydroxyl group of HAp [22,23,25]. Because ZOL/β-TCP powders mainly consisted of two components (β-TCP and HAp) (Figure 1), we hypothesized that ZOL/β-TCP would bind via both chelation and electrostatic interactions. The authors defined the two binding states of chelation and electrostatic interactions as higher or lower (or loose) binding affinity, respectively [23]. However, the binding force was not indicated. 

ZOL/β-TCP slightly released ZOL (Figure 3a,b). Most ZOL remained on the β-TCP disc, and a part of ZOL with lower binding affinity was released from ZOL/β-TCP. In addition, ZOL was further released from ZOL/β-TCP under the condition of activating osteoclasts. Activated osteoclasts create weak acid conditions locally with the resorption of bone minerals. At pH 5.5, ZOL can bind to CaP, although the ZOL binding affinity to CaP at pH 5.5 was lower than that at pH 7.3 [41]. In this study, the change in the zeta potential of ZOL/β-TCP suggested that the binding state of ZOL to β-TCP was changed on the basis of the surrounding pH (Table 1). The p*K*_a_ values of the R2 functional group were particularly related to the changes in the bound state. The p*K*_a_ value of the imidazole ring—the R2 functional group—in ZOL was 6.953. At pH 5.5, the imidazole ring remained protonated, and the zeta potential changed positively. However, at pH 7.3, the deprotonation of the imidazole ring reduced the positive charges. Additionally, the phosphonate group of ZOL (p*K*_a1_ = 2.89, p*K*_a2_ = 6.63) decreased the positive charge by deprotonating at pH 7.3 [42], which shifted the zeta potential negatively. In contrast, the phosphate group in ZOL was protonated at pH 5.5 [42], which is responsible for shifting the zeta potential positively and decreasing the binding force of ZOL to β-TCP. Therefore, osteoclast-mediated resorption can create weak acid conditions on the surface of ZOL/β-TCP. ZOL is expected to be further released.

The rate of ZOL release from ZOL/β-TCP powders and disc decreased with an increase in loading ZOL on β-TCP (data not shown). The increase in loading ZOL induces the formation of an interaction between ZOL and ZOL. As mentioned earlier, binding of N-BPs to CaP enables the attraction of more N-BPs on the surface of CaP via electrostatic interactions. In addition, the Ca^2+^ dissolution rate of ZOL/β-TCP showed behavior similar to that of released ZOL. (Figure 2). The strong binding affinity of N-BPs to Ca^2+^ inhibits Ca^2+^ dissolution, which in turn inhibits the conversion of CaP into HAp [23,28]. We also confirmed that HAp conversion by ZOL/β-TCP was inhibited (Figure 1). Therefore, binding ZOL to β-TCP can control the Ca^2+^ dissolution and ZOL release due to the change in surface charge of β-TCP and ZOL. 

The weak interactions between ZOL and β-TCP release ZOL, and the released ZOL binds Ca^2+^ on β-TCP again [27,41]. ZOL, which has a high affinity for Ca^2+^, would be poorly diffused and stays close to accessible surfaces [41]. On dentin disc coatings with fluorescent labeling alendronate (FL-ALN), relabeling was observed on the newly exposed surfaces of resorption pits using “recycled” FL-ALN [43]. Similar phenomenon, recycled ZOL, would occur in this study. Moreover, in this examination, the capacity to bind ZOL to β-TCP was sufficient, and a greater amount of ZOL bounded to ZOL/β-TCP. Therefore, ZOL/β-TCP sustainably affected target cells during the recycling of ZOL. 

The material properties indicate that ZOL can be released from ZOL/β-TCP and that it can work in target cells. In addition, local and sustainable treatments can be enabled by abiding surrounding the target region. The proliferation and activation of the target cells were inhibited by ZOL(1.5, 3, 6)/β-TCP via both bound and free-ZOL (Figure 4a, Figure 5a, Figure 6, Figure 7, Figure 8 and Figure 9). Intercellular Ca^2+^ supports the uptake of N-BPs to osteoclasts [44]. Bound-ZOL is synergistically taken up by osteoclasts by binding ZOL to Ca^2+^. Similar effects of Ca^2+^ on tumor cells have been reported [45]. Therefore, the combination of Ca^2+^ and ZOL is useful for the treatment of osteosarcoma. In addition, CM can be affected by osteoclasts (Figure 8 and Figure 9). The data showed that ZOL was released by osteoclast-mediated resorption. However, CM(3) was found to be the most effective, even though osteoclasts were not activated on ZOL(3)/β-TCP. The results showed that ZOL/β-TCP was released by contacting cells. In addition, the ZOL on ZOL(3)/β-TCP disc was double the amount of ZOL on ZOL(1.5)/β-TCP discs. Therefore, the weak acid condition was created by a slight number of activated-osteoclasts until day 5 (DAY5, Figure 6 and Figure 7). This condition enabled the release of ZOL. The actual release behavior in the culture medium is unknown. However, we confirmed that both bound and free-ZOL express the inhibitory effects for target cells, and the activated-osteoclasts further release the ZOL. The two states of ZOL enable local and sustainable treatment. 

ZOL(1.5, 3)/β-TCP did not inhibit osteoblast viability on days 1 and 3 (Figure 4b and Figure 5b). As previously reported, N-BPs decreased osteoblast survival rate [46,47]. The reason for the non-inhibition of osteoblast viability by ZOL/β-TCP is attributed to the involvement of Ca^2+^ derived from β-TCP. Extracellular Ca^2+^ promotes mesenchymal stromal cell (MSC) proliferation and differentiation [48]. However, in our study, it was observed that, neither proliferative activation, nor inhibition of osteoblast proliferation, was exhibited by ZOL(0, 1.5, 3)/β-TCP. Moreover, the detailed causes for this behavior were not found.

Additionally, we expected additional effects of ZOL/β-TCP on the treatment of osteosarcoma. N-BPs, including ZOL, are reported to have anti-angiogenic effects by inhibiting the survival and migration of vascular endothelial and tumor cells [49,50]. In addition, ZOL was previously reported to inhibit angiogenesis by inhibiting preosteoclast differentiation. ZOL inhibits the secretion of PDGF-BB from preosteoclasts [51]. PDGF-BB is a factor promoting angiogenesis and is supplied through osteoclast differentiation [51,52]. We hypothesized that ZOL/β-TCP would inhibit angiogenesis by inhibiting the proliferation of vascular endothelial cells and the proliferation and differentiation of tumor cells and osteoclasts. However, additional examination is necessary to validate the anti-angiogenic effects of ZOL/β-TCP.

In this study, we confirmed that ZOL/β-TCP can hold/release the target region locally and sustainably via bound and free-ZOL. In addition, we demonstrated that ZOL/β-TCP can inhibit the proliferation and differentiation of tumor cells and osteoclasts. ZOL, which binds to β-TCP and is subsequently released from the ZOL/β-TCP, prevents relapse by destroying the vicious cycle between tumor cells and osteoclasts. These data revealed that ZOL/β-TCP would be useful for treating osteosarcoma.

## 4. Materials and Methods

### 4.1. Sample Fabrication and Characterization

#### 4.1.1. Preparation of β-TCP Powders and Fabrication of Discs

Commercially-available β-tricalcium phosphate (β-TCP-100, Taihei Chemical Industrial Co. Ltd., Osaka, Osaka, Japan) powders (10 g) was ground using a planetary mill (Pulverisette 6, Fritch Japan Co. Ltd., Yokohama, Kanagawa, Japan) for 180 min at 300 rpm in a ZrO_2_ beads (180 g) with 2 mm diameter in 40 mL of ultrapure water. After ball-milling, the slurry was filtered and freeze-dried for 24 h. In addition, to fabricate discs, each powder (0.1 g) was mixed with 2.5 mass% Na_2_HPO_4_ (Wako Pure Chemical, Osaka, Osaka, Japan) solution (25 μL) and uniaxially compressed at 20 MPa to form compacts (*φ* = 10 mm).

#### 4.1.2. Adsorption of Zoledronic acid to β-TCP Powders

Zoledronic acid (ZOL; Zoledronic Acid Monohydrate, Tokyo Chemical Industry Co. Ltd., Tokyo, Japan) solutions (0, 1.5, 3.0, 6.0 mmol/L) were adjusted at pH 7.3 by NaOH (Wako Pure Chemical, Osaka, Osaka, Japan) solutions and diluted by ultrapure water. β-TCP powders (1.5 g) was added into ZOL solutions (40 mL) and rotated at 60 rpm for 1 h at 25 °C. The samples were centrifuged at 8000 rpm for 10 min. The powders were washed with ultrapure water (40 mL) and centrifuged at 8000 rpm for 10 min. The supernatant was used to determine concentration of loaded the ZOL to β-TCP powders by measuring the optical density at 217 nm with absorption photometer (GENEQUANT 100, biochrom, Harvard Bioscience, Inc., Holliston, MA, USA); and the powders were freeze-dried. Hereafter, the resulting powders are donated by the ZOL(X)/β-TCP; X represents the concentration of ZOL solution. Loaded ZOL was calculated by Equation (1). 


Loaded ZOL (mass%) = Adsorbed ZOL (g)/β-TCP (g)
(1)

#### 4.1.3. X-ray Diffraction Analysis

X-ray diffraction (XRD) patterns of the ZOL/β-TCP powders were determined using an X-ray diffractometer (Ultima IV, Rigaku Co., Akishima, Tokyo, Japan) equipped with a Cu Kα radiation source. Date were collected in the range 2θ = 10 to 50°, with a step size of 0.02° and counting time of 1.2 s/step. The tube current was 40 mA with a tube voltage of 40 kV. The crystalline phases were identified using the International Center for Diffraction Date-Powder Diffraction File PDF (ICDDPDF) for β-TCP (#09-0169) and Hydroxyapatite (HAp) (#09-0432).

#### 4.1.4. Zeta Potential 

Zeta potentials of the ZOL/β-TCP powders measured using a laser-doppler velocimeter (ELS-6000, Otsuka Electronics, Hirakata, Osaka, Japan). Each ZOL/β-TCP powder was suspended in 0.01 mol/L NaCl (Wako Pure Chemical, Osaka, Osaka, Japan) solution (50 mL); and all zeta potentials were measured while controlled at pH 5.5 or 7.3 by NaOH solution and HCl (Wako Pure Chemical, Osaka, Osaka, Japan) solution. 

#### 4.1.5. Solubility of Ca^2+^ from ZOL/β-TCP Powders

Solubility of Ca^2+^ from the ZOL/β-TCP powders was determined using Benchtop water quality meters (LAQUA F-73, HORIBA, LTD., Kyoto, Kyoto, Japan) at pH 5.5 and 7.3. To compare with that of HAp, we also used HAp powders (HAP-100, Taihei Chemical Industrial Co. Ltd., Osaka, Osaka, Japan). Each ZOL/β-TCP powder (0.025 g) was stirred at 430 rpm in Acetate buffer (pH 5.5, 0.08 mol/L) or Tris-HCl buffer (pH 7.3, 0.05 mol/L) for 3 h at 25 °C. A dissolution rate of Ca^2+^ from ZOL/β-TCP powders was calculated in accordance with JIST0330-3.

#### 4.1.6. Release of ZOL from ZOL/β-TCP Powders and ZOL/β-TCP Disc

The release tests of ZOL were examined on the ZOL/β-TCP powders and ZOL/β-TCP disc. The ZOL/β-TCP powders (0.1 g) were immersed in 10 mL Tris-HCl buffer (pH 7.3, 0.05 mol/L) for the desired period at 25 °C. Buffer (5 mL) was changed before the measurement (at days 1, 3, 5, and 7). Discs (10 mm*φ*) were set on polystyrene plates, which were filled with 1 mL Tris-HCl buffer (pH 7.3, 0.05 mol/L) for 1, 3 days at 37 °C in an incubator. 

The concentration of ZOL in the supernatant was measured by using the quantitative method described in the literature [53]. Concentration of complex formation with the ZOL and Fe^3+^ were determined by measuring the optical density at 290 nm with absorbance photometer (GENEQUANT 100, biochrom, Harvard Bioscience, Inc., Holliston, MA, USA).

### 4.2. Biological Evaluation

#### 4.2.1. Cell Culture and Isolation 

For biological evaluation of ZOL/β-TCP, MC3T3-E1, HOS, and mice bone marrow stromal cells (mBMSCs) as osteoblasts, tumor cells, and osteoclasts were used in this study. Every cell was cultured under a humidified atmosphere of 5% at 37 °C. 

MC3T3-E1 were cultured in Alpha modified Eagle’s medium (α-MEM, Gibco, Thermo Fisher Scientific, Waltham, MA, USA) with heat inactivated fetal bovine serum (FBS, Wako Pure Chemical, Osaka, Japan) and 0.1% antibiotics (100 U/mL penicillin and 100 μg/mL streptomycin solution, Wako Pure Chemical, Osaka, Osaka, Japan). HOS were cultured in Eagle’s minimum modified medium (EMEM, Sigma-Aldrich, St. Louis, MO, USA) with 1% non-Essential amino acids (Sigma-Aldrich, St. Louis, MO, USA) and 10% FBS and 0.1% antibiotics. 

Nine to ten weeks-old C57BL/6 male mice (C57BL/6NCrSlc, Japan SLC, Inc., Hamamatsu, Shizuoka, Japan) were sacrificed, and mBMSCs were isolated from the bone marrow. mBMSCs were cultured on non-adhesive dish in α-MEM with 10% FBS and 0.1% antibiotics for 24 h at 37 °C. After that, non-adhesive cells were recovered from supernatant culture medium and cultured in α-MEM with 10% FBS and 0.1% antibiotics and 50 ng/mL macrophage colony-stimulating factor (MCS-F, Peprotech Inc., Cranbury, NJ, USA) for 24 h. The supernatant medium was removed and the loosely-adhesive cells were recovered by suspending of pipet and used for in vitro test of osteoclasts. All of the animal treatments were approved by the Animal Research Committee of Meiji University (MUIACUC2020-12 (5th/June/2020)). 

#### 4.2.2. Preparation of Bone Slices

To prepare bone slice, nine to ten weeks-old C57BL/6 male mice (C57BL/6NCrSlc, Japan SLC, Inc., Hamamatsu, Shizuoka, Japan) was sacrificed, and about 5 mm-diameter circle calvaria bone was cut off from each side of the calvaria. Bone slices were washed with phosphate-buffered saline (PBS) containing 0.1% triton X-100 (Wako Pure Chemical, Osaka, Osaka, Japan) several times. After washing, bone slice was stored in 70% ethanol at −20 °C. Before using for culture, the thawed bone slice washed PBS and immerged in α-MEM. 

#### 4.2.3. Preparation of Elution Medium

To examine of the effect of bound-ZOL, we used ZOL/β-TCP disc. Each ZOL/β-TCP disc fabricated in accordance with the process of 4.1.1 was sterilized by dry heat. To examine of the effect of free-ZOL, each cell was cultured in an elution medium. Each elution medium of ZOL(X)/β-TCP (EM(X)) was adjusted by immersing of each ZOL/β-TCP disc in culture medium (1 mL) and incubating for 24 h at 37 °C. After incubating, each supernatant medium was collected and centrifuged at 3000 rpm for 5 min.

#### 4.2.4. Cell Viability of MC3T3-E1 and HOS

Cell viability of MC3T3-E1 and HOS were determined by a 3-(4, 5-Dimethylthiazol-2-yl)-2,5-diphenyltetrazoloum bromide (MTT) assay. At first, to investigate of the effect of bound-ZOL between each cell and ZOL/β-TCP, MC3T3-E1 (5.0 × 10^5^ cells/well, passage 3–10) and HOS (1.0 × 10^5^ cells/well, passage 3–10) were cultured on each ZOL/β-TCP disc for 24, 72 h. In this examination, we used the ZOL/β-TCP disc immersing in culture medium for 24 h before seeding each cell. In addition, to examine the effect of free-ZOL, MC3T3-E1 (5.0 × 10^4^ cells/well, passage 3–10) and HOS (1.0 × 10^4^ cells/well, passage 3–10) were cultured on 48-well plate in each 1 mL EM for 24, 72 h. After the indicated culture time in each experiment, MTT solution (5 mg/mL, Dojindo, Kumamoto, Japan) was added and incubated at 37 °C for 4 h. Then, the culture medium was removed and MTT formazan crystals were resolved in 300 μL dimethyl sulfoxide (DMSO, Wako Pure Chemical, Osaka, Japan). The absorbances were measured at 570 nm (measurement wavelength) and 650 nm (reference wavelength) using a microplate reader (Thermo Fisher Scientific, Waltham, MA, USA). The viability of each cell was calculated by the ratio between the absorbance of culturing on the ZOL(0)/β-TCP disc or in EM(0) and the absorbance of culturing on the ZOL(1.5, 3, 6)/β-TCP discs or in EM(1.5, 3, 6). 

#### 4.2.5. Culture of mBMSCs on each ZOL/β-TCP Disc

To examine the effects of the ZOL/β-TCP for osteoclast formation and activation, mBMSCs were cultured on each ZOL/β-TCP disc in accordance with the schedule of Figure 6. In brief, isolated mBMSCs (5.0 × 10^5^ cells/well) were cultured on ZOL/β-TCP discs in differentiation inducer medium, α-MEM with 10% FBS, 0.1% antibiotics, 30 ng/mL M-CSF and 50 ng/mL receptor for nuclear factor kappa B ligand (RANKL, Peprotech Inc., Cranbury, NJ, USA), for 5, 10 days at 37 °C. In this examination, we used the ZOL/β-TCP disc, immersing in culture medium for 24 h before seeding mBMSCs. The medium was exchanged every 2, 3 days. After culturing, to confirm osteoclast formation, cells were stained with Alexa Fluor^®^ 488-labeled phalloidin (Invitrogen, Carlsbad, CA, USA) for F-actin and DAPI (Dojindo, Kamimashiki, Kumamoto, Japan) for nuclei. Cells were washed with PBS (pH 7.3) and examined by observation with fluorescence microscopy (BZ-X710, Keyence, Osaka, Osaka, Japan). To confirm osteoclast activation, cells were performed with tartrate-resident acid phosphate (TRAP) staining using TRAP/ALP Stain Kit (Wako Pure Chemical, Osaka, Japan) and observed by phase microscopy (BZ-X710, Keyence, Osaka, Osaka, Japan). We analyzed three indexes from the results of observation: the osteoclasts area, cells size, and TRAP-positive area by BZ-X Analyzer (BZ-H3A (ver. 1.31), Keyence, Osaka, Osaka, Japan). 

#### 4.2.6. Preparation of Osteoclastic Conditioned Medium

To investigate of the effect of free-ZOL, we prepared osteoclastic (on ZOL(X)/β-TCP disc) conditioned medium (CM(X)). The culture medium obtained when was exchanging of mBMSCs culturing on each ZOL/β-TCP disc (above culture of mBMSCs (4.2.5)) was recovered and centrifuged at 3000 rpm for 5 min. After that, 30 ng/mL MCS-F and 50 ng/mL RANKL were added in the recovered medium. 

#### 4.2.7. Culture of mBMSCs in an Osteoclastic Conditioned Medium

We seeded isolated mBMSCs (5.0 × 10^4^ cells/well) on a 48-well plate or bone slice and cultured in differentiation inducer medium for 5 days. We treated osteoclasts with each 1 mL CM on the 48 well-plate for 3 days or on the bone slice for 5 days. After culturing, we stained the nuclei, F-actin, and TRAP positive cells of osteoclasts on a 48-well plate. The effects of CM were examined by observation with fluorescence microscopy (BZ-X710, Keyence, Osaka, Osaka, Japan). We analyzed the TRAP-positive area from the results of observation using a BZ-X Analyzer (BZ-H3A (ver. 1.31), Keyence, Osaka, Osaka, Japan). The cells on the bone slice were observed with scanning electron microscopy (VE9800, Keyence, Osaka, Japan).

### 4.3. Statistical Analysis

All experiments were repeated at least three times independently. Data were statistically analyzed to determine the mean and the standard deviation of the mean. Differences between groups were tested by Student’s *t* test. A value of *p* < 0.05 was considered significant.

## 5. Conclusions

We proposed ZOL, which is an anti-tumor and anti-resorptive agent, and proposed a novel material that can be used for the local administration of ZOL. In this study, we developed a new carrier (ZOL/β-TCP) for delivering the ZOL to the target region. Additionally, we evaluated its potential in vitro. Our data show that ZOL/β-TCP can release ZOL locally and sustainably and further released ZOL by osteoclast-mediated resorption of ZOL/β-TCP. Moreover, ZOL/β-TCP exerted inhibitory effects on tumor cells and osteoclasts via bound-ZOL (binding to β-TCP) and free-ZOL (release from ZOL/β-TCP). These results showed that ZOL/β-TCP is expected to prevent relapse and metastasis due to delivery of the ZOL locally and sustainably and will be a useful material for treatment of osteosarcoma in lieu of existing bone substitutes. 

## Figures and Tables

**Table 1:** Characterization of zoledronic acid (ZOL)/β-tricalcium phosphate (β-TCP) powders.

Sample	Charged-ZOL	Loaded-ZOL	Zeta Potential	
	(mmol/L)	(mass%)	pH 7.3	pH 5.5
			(mV)	(mV)
ZOL(0)	0	0.00	−9.46 ± 1.76	−12.25 ± 2.41
ZOL(1.5)	1.5	1.07 ± 0.0422	−10.44 ± 2.51	−9.13 ± 1.49
ZOL(3)	3	2.11 ± 0.106	−12.20 ± 1.50	−6.19 ± 1.94
ZOL(6)	6	4.28 ± 0.0882	−14.81 ± 1.77	−0.96 ± 2.30

## Data Availability

Data available in a publicly accessible repository.

## Supplementary Materials

The following are available online at https://www.mdpi.com/1422-0067/22/4/1889/s1, Figure S1: Molecular structure, Figure S2: ZOL binding affinity to β-TCP powders, Figure S3: Morphological observation of cells on each ZOL/β-TCP disc, Figure S4: Morphological observation of cells in elution medium of ZOL/β-TCP, Figure S5: Cell adhesion of mBMSCs on ZOL/β-TCP disc, Figure S6: Observation of mBMSCs and pit area, Figure S7: Morphological observation of osteoclasts and apoptotic bodies in osteoclastic conditioned medium.

## References

[1] Mirabello L., Troisi R.J., Savage S.A. (2009). International osteosarcoma incidence patterns in children and adolescents, middle ages and elderly persons. Int. J. Cancer.

[2] Picci P. (2007). Osteosarcoma (osteogenic sarcoma). Orphanet J. Rare Dis..

[3] Ponzetti M., Rucci N. (2020). Switching Homes: How Cancer Moves to Bone. Int. J. Mol. Sci..

[4] Zheng Y., Zhou H., Dunstan C.R., Sutherland R.L., Seibel M.J. (2013). The role of the bone microenvironment in skeletal metastasis. J. Bone Oncol..

[5] Clines G.A., Guise T.A. (2008). Molecular mechanisms and treatment of bone metastasis. Expert Rev. Mol. Med..

[6] Soeharno H., Povegliano L., Choong P.F. (2018). Multimodal treatment of bone metastasis—A surgical perspective. Front. Endocrinol..

[7] Meyers P.A., Heller G., Healey J.H., Huvos A., Applewhite A., Sun M., LaQuaglia M. (1993). Osteogenic sarcoma with clinically detectable metastasis at initial presentation. J. Clin. Oncol..

[8] Chou A.J., Merola P.R., Wexler L.H., Gorlick R.G., Vyas Y.M., Healey J.H., LaQuaglia M.P., Huvos A.G., Meyers P.A. (2005). Treatment of osteosarcoma at first recurrence after contemporary therapy: The Memorial Sloan-Kettering Cancer Center experience. Cancer Interdisciplinary Int. J. Am. Cancer Soc..

[9] Van Beek E., Pieterman E., Cohen L., Löwik C., Papapoulos S. (1999). Farnesyl pyrophosphate synthase is the molecular target of nitrogen-containing bisphosphonates. Biochem. Biophys. Res. Commun..

[10] Guo R.-T., Cao R., Liang P.-H., Ko T.-P., Chang T.-H., Hudock M.P., Jeng W.-Y., Chen C.K.-M., Zhang Y., Song Y. (2007). Bisphosphonates target multiple sites in both cis-and trans-prenyltransferases. Proc. Natl. Acad. Sci. USA.

[11] Fleisch H. (2007). Introduction to bisphosphonates. History and functional mechanisms. Der Orthop..

[12] Drake M.T., Clarke B.L., Khosla S. (2008). Mayo Clinic Proceedings.

[13] Green J.R. (2003). Antitumor effects of bisphosphonates. Cancer Interdiscip. Int. J. Am. Cancer Soc..

[14] Green J.R. (2004). Bisphosphonates: Preclinical review. Oncologist.

[15] Ravn P., Neugebauer G., Christiansen C. (2002). Association between pharmacokinetics of oral ibandronate and clinical response in bone mass and bone turnover in women with postmenopausal osteoporosis. Bone.

[16] Hadji P., Coleman R.E., Wilson C., Powles T., Clézardin P., Aapro M., Costa L., Body J.-J., Markopoulos C., Santini D. (2016). Adjuvant bisphosphonates in early breast cancer: Consensus guidance for clinical practice from a European Panel. Ann. Oncol..

[17] Reyes C., Hitz M., Prieto-Alhambra D., Abrahamsen B. (2016). Risks and benefits of bisphosphonate therapies. J. Cell. Biochem..

[18] Lacerna L., Hohneker J. (2003). Zoledronic acid for the treatment of bone metastases in patients with breast cancer and other solid tumors. Semin. Oncol..

[19] Verron E., Bouler J. (2014). Is bisphosphonate therapy compromised by the emergence of adverse bone disorders?. Drug Discov. Today.

[20] Verron E., Khairoun I., Guicheux J., Bouler J.-M. (2010). Calcium phosphate biomaterials as bone drug delivery systems: A review. Drug Discov. Today.

[21] Boanini E., Gazzano M., Bigi A. (2012). Time course of zoledronate interaction with hydroxyapatite nanocrystals. J. Phys. Chem. C.

[22] Boanini E., Torricelli P., Gazzano M., Fini M., Bigi A. (2012). The effect of zoledronate-hydroxyapatite nanocomposites on osteoclasts and osteoblast-like cells in vitro. Biomaterials.

[23] Forte L., Torricelli P., Boanini E., Gazzano M., Fini M., Bigi A. (2017). Antiresorptive and anti-angiogenetic octacalcium phosphate functionalized with bisphosphonates: An in vitro tri-culture study. Acta Biomater..

[24] Pan H.-B., Darvell B. (2009). Calcium phosphate solubility: The need for re-evaluation. Cryst. Growth Des..

[25] Russell R., Watts N., Ebetino F., Rogers M. (2008). Mechanisms of action of bisphosphonates: Similarities and differences and their potential influence on clinical efficacy. Osteoporos. Int..

[26] Sato M., Grasser W., Endo N., Akins R., Simmons H., Thompson D., Golub E., Rodan G. (1991). Bisphosphonate action. Alendronate localization in rat bone and effects on osteoclast ultrastructure. J. Clin. Investig..

[27] Nancollas G.H., Tang R., Phipps R.J., Henneman Z., Gulde S., Wu W., Mangood A., Russell R.G.G., Ebetino F.H. (2006). Novel insights into actions of bisphosphonates on bone: Differences in interactions with hydroxyapatite. Bone.

[28] Shen Z., Yu T., Ye J. (2014). Microstructure and properties of alendronate-loaded calcium phosphate cement. Mater. Sci. Eng. C.

[29] Garay T., Kenessey I., Molnár E., Juhász É., Réti A., László V., Rózsás A., Dobos J., Döme B., Berger W. (2015). Prenylation inhibition-induced cell death in melanoma: Reduced sensitivity in BRAF mutant/PTEN wild-type melanoma cells. PLoS ONE.

[30] Tai T.-W., Chen C.-Y., Su F.-C., Tu Y.-K., Tsai T.-T., Lin C.-F., Jou I.-M. (2017). Reactive oxygen species are required for zoledronic acid-induced apoptosis in osteoclast precursors and mature osteoclast-like cells. Sci. Rep..

[31] Junrui P., Bingyun L., Yanhui G., Xu J., Darko G.M., Dianjun S. (2016). Relationship between fluoride exposure and osteoclast markers during RANKL-induced osteoclast differentiation. Environ. Toxicol. Pharmacol..

[32] Zhou H., Chernecky R., Davies J. (1993). Scanning electron microscopy of the osteoclast-bone interface in vivo. Cells Mater..

[33] Balvan J., Krizova A., Gumulec J., Raudenska M., Sladek Z., Sedlackova M., Babula P., Sztalmachova M., Kizek R., Chmelik R. (2015). Multimodal holographic microscopy: Distinction between apoptosis and oncosis. PLoS ONE.

[34] Santini D., Schiavon G., Angeletti S., Vincenzi B., Gasparro S., Grilli C., La Cesa A., Virzi V., Leoni V., Budillon A. (2006). Last generation of amino-bisphosphonates (N-BPs) and cancer angiogenesis: A new role for these drugs?. Recent Pat. Anti-Cancer Drug Discov..

[35] Chen J., Ashames A., Buabeid M.A., Fahelelbom K.M., Ijaz M., Murtaza G. (2020). Nanocomposites drug delivery systems for the healing of bone fractures. Int. J. Pharm..

[36] Eddy A., Tsuchiya K., Tsuru K. (2018). Ishikawa, Fabrication of self-setting β-TCP granular cement using β-TCP granules and sodium hydrogen sulfate solution. J. Biomater. Appl..

[37] Mariño F.T., Torres J., Tresguerres I., Jerez L.B., Cabarcos E.L. (2007). Vertical bone augmentation with granulated brushite cement set in glycolic acid. J. Biomed. Mater. Res. Part A.

[38] Kharazmi M., Persson U., Warfvinge G. (2012). Pharmacovigilance of oral bisphosphonates: Adverse effects manifesting in the soft tissue of the oral cavity. J. Oral Maxillofac. Surg..

[39] Lanza F.L., Hunt R.H., Thomson A.B., Provenza J.M., Blank M.A., Group R.E.S. (2000). Endoscopic comparison of esophageal and gastroduodenal effects of risedronate and alendronate in postmenopausal women. Gastroenterology.

[40] Sharma D., Ivanovski S., Slevin M., Hamlet S., Pop T.S., Brinzaniuc K., Petcu E.B., Miroiu R.I. (2013). Bisphosphonate-related osteonecrosis of jaw (BRONJ): Diagnostic criteria and possible pathogenic mechanisms of an unexpected anti-angiogenic side effect. Vasc. Cell.

[41] Russell R.G.G. (2007). Determinants of structure–function relationships among bisphosphonates. Bone.

[42] Nishiguchi A., Taguchi T. (2019). Osteoclast-responsive, injectable bone of bisphosphonated-nanocellulose that regulates osteoclast/osteoblast activity for bone regeneration. Biomacromolecules.

[43] Coxon F.P., Thompson K., Roelofs A.J., Ebetino F.H., Rogers M.J. (2008). Visualizing mineral binding and uptake of bisphosphonate by osteoclasts and non-resorbing cells. Bone.

[44] Thompson K., Rogers M.J., Coxon F.P., Crockett J.C. (2006). Cytosolic entry of bisphosphonate drugs requires acidification of vesicles after fluid-phase endocytosis. Mol. Pharmacol..

[45] Merrell M.A., Wakchoure S., Ilvesaro J.M., Zinn K., Gehrs B., Lehenkari P.P., Harris K.W., Selander K.S. (2007). Differential effects of Ca2+ on bisphosphonate-induced growth inhibition in breast cancer and mesothelioma cells. Eur. J. Pharmacol..

[46] Huang X., Huang S., Guo F., Xu F., Cheng P., Ye Y., Dong Y., Xiang W., Chen A. (2016). Dose-dependent inhibitory effects of zoledronic acid on osteoblast viability and function in vitro. Mol. Med. Rep..

[47] Scala R., Maqoud F., Angelelli M., Latorre R., Perrone M.G., Scilimati A., Tricarico D. (2019). Zoledronic acid modulation of TRPV1 channel currents in osteoblast cell line and native rat and mouse bone marrow-derived osteoblasts: Cell proliferation and mineralization effect. Cancers.

[48] Lee M.N., Hwang H.-S., Oh S.-H., Roshanzadeh A., Kim J.-W., Song J.H., Kim E.-S., Koh J.-T. (2018). Elevated extracellular calcium ions promote proliferation and migration of mesenchymal stem cells via increasing osteopontin expression. Exp. Mol. Med..

[49] Misso G., Porru M., Stoppacciaro A., Castellano M., De Cicco F., Leonetti C., Santini D., Caraglia M. (2012). Evaluation of the in vitro and in vivo antiangiogenic effects of denosumab and zoledronic acid. Cancer Biol. Ther..

[50] Zafar S., Cullinan M.P., Drummond B.K., Seymour G.J., Coates D.E. (2020). Effects of zoledronic acid and geranylgeraniol on angiogenic gene expression in primary human osteoclasts. J. Oral Sci..

[51] Gao S.-Y., Zheng G.-S., Wang L., Liang Y.-J., Zhang S.-E., Lao X.-M., Li K., Liao G.-Q. (2017). Zoledronate suppressed angiogenesis and osteogenesis by inhibiting osteoclasts formation and secretion of PDGF-BB. PLoS ONE.

[52] Rahman M.M., Matsuoka K., Takeshita S., Ikeda K. (2015). Secretion of PDGF isoforms during osteoclastogenesis and its modulation by anti-osteoclast drugs. Biochem. Biophys. Res. Commun..

[53] Kuljanin J., Janković I., Nedeljković J., Prstojević D., Marinković V. (2002). Spectrophotometric determination of alendronate in pharmaceutical formulations via complex formation with Fe (III) ions. J. Pharm. Biomed. Anal..

