# Supplementary material for: Zoledronic Acid-Loaded β-TCP Inhibits Tumor Proliferation and Osteoclast Activation: Development of a Functional Bone Substitute for an Efficient Osteosarcoma Treatment
Source: Int J Mol Sci. 2021 Feb 14;22(4):1889. doi: 10.3390/ijms22041889 (PMC7918630; doi:10.3390/ijms22041889)
Supplement: Supplementary file 1 [file ijms-22-01889-s001.pdf]

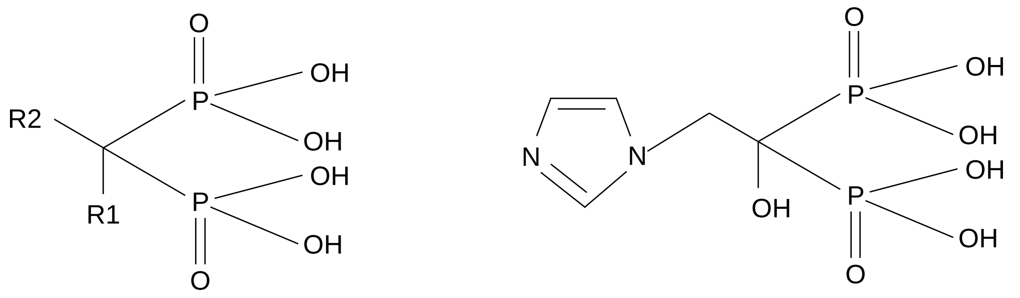

Bisphosphonate

Zoledronic acid (ZOL)

Figure S1. Molecular structure.
(a) Chemical basic structure of BPs, (b) Chemical structure of ZOL.

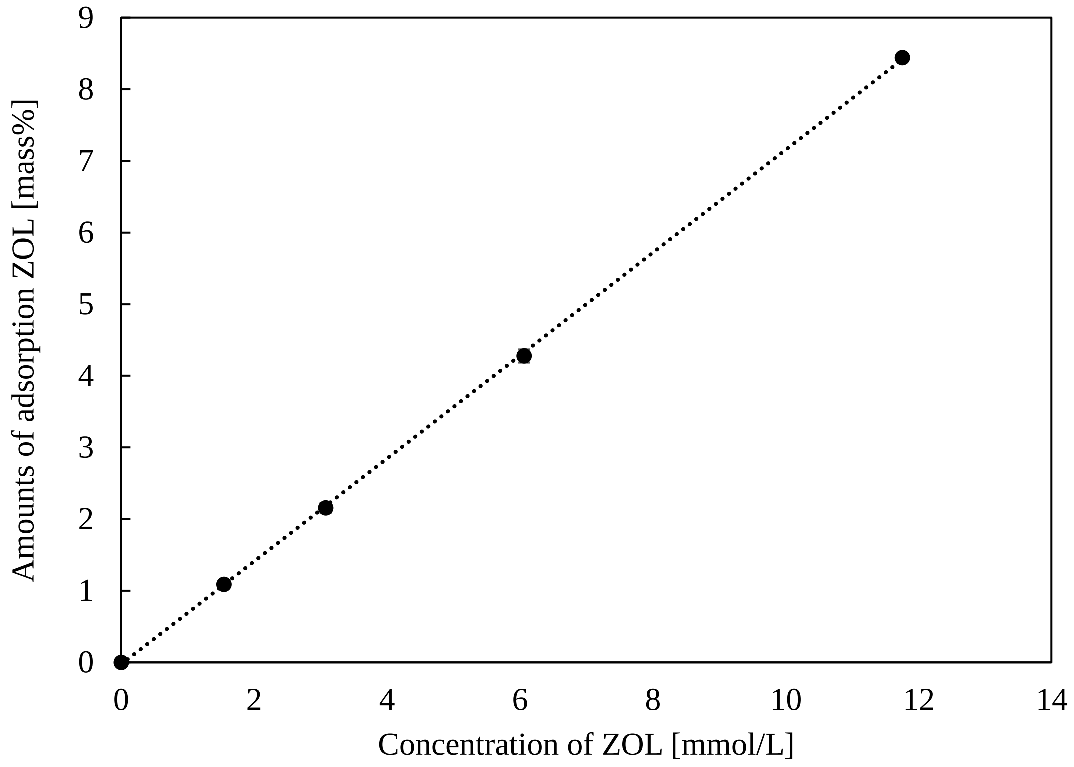

Figure S2. ZOL binding affinity to β-TCP powders.
β-TCP powders were rotated with each ZOL solution (0, 1.5, 3, 6, 12 mmol/L) for 1 h and loaded ZOL. The straight line was showed adsorption level of the ZOL to β-TCP powders. Error bars indicate standard deviation of the mean.

(a)

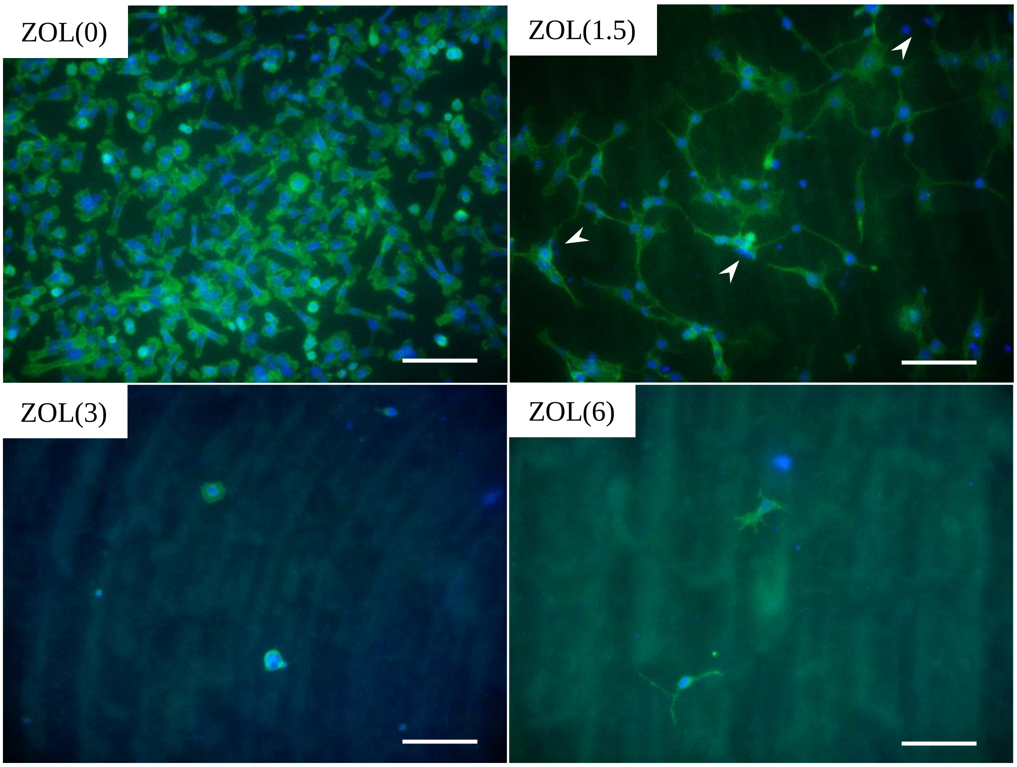

(b)

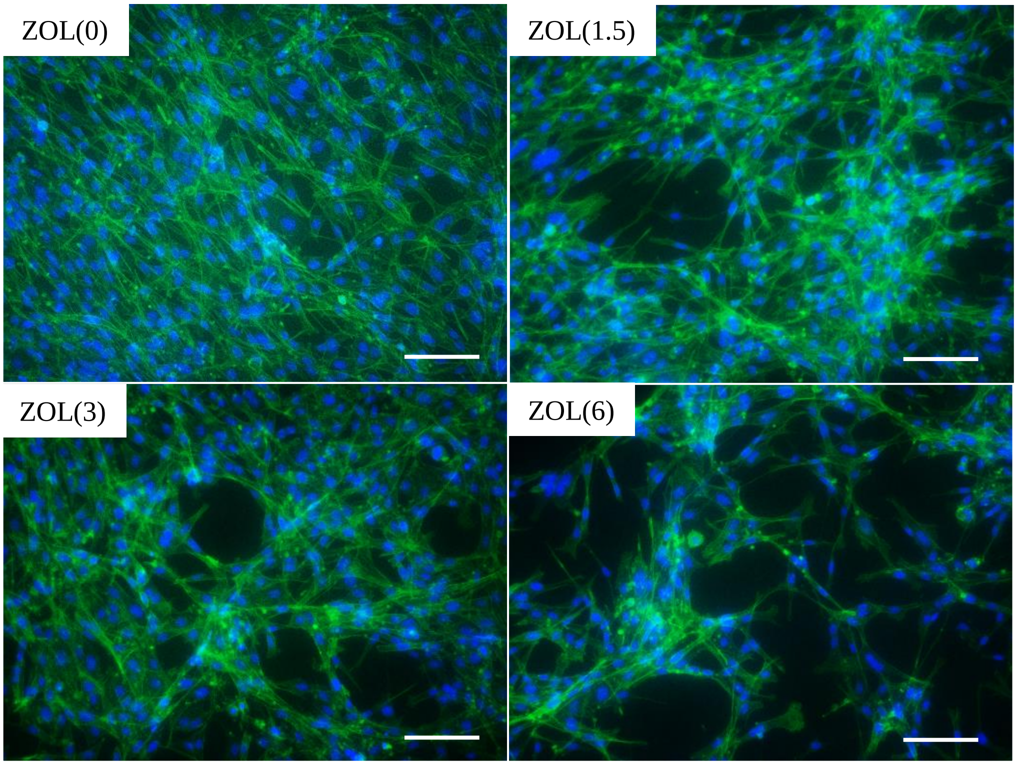

Figure S3. Morphological observation of cells on each ZOL/β-TCP disc.

HOS and MC3T3-E1 were cultured on each ZOL/β-TCP disc for 3 days. After culturing, (a) HOS and (b) MC3T3-E1 were stained with Alexa Flour® 488-labeled phalloidin for F-actin (green) and DAPI for nuclei (blue) and observed with fluorescence microscopy. Scale bars: 100 μm, arrow heads; apoptotic-like morphology.

(a)

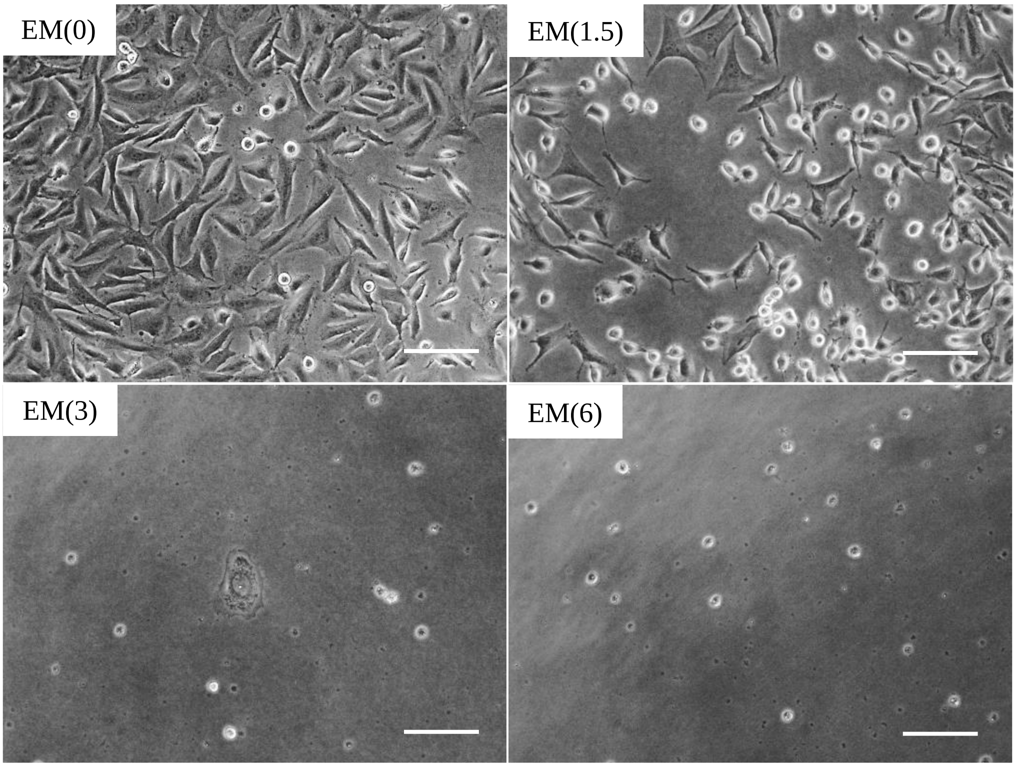

(b)

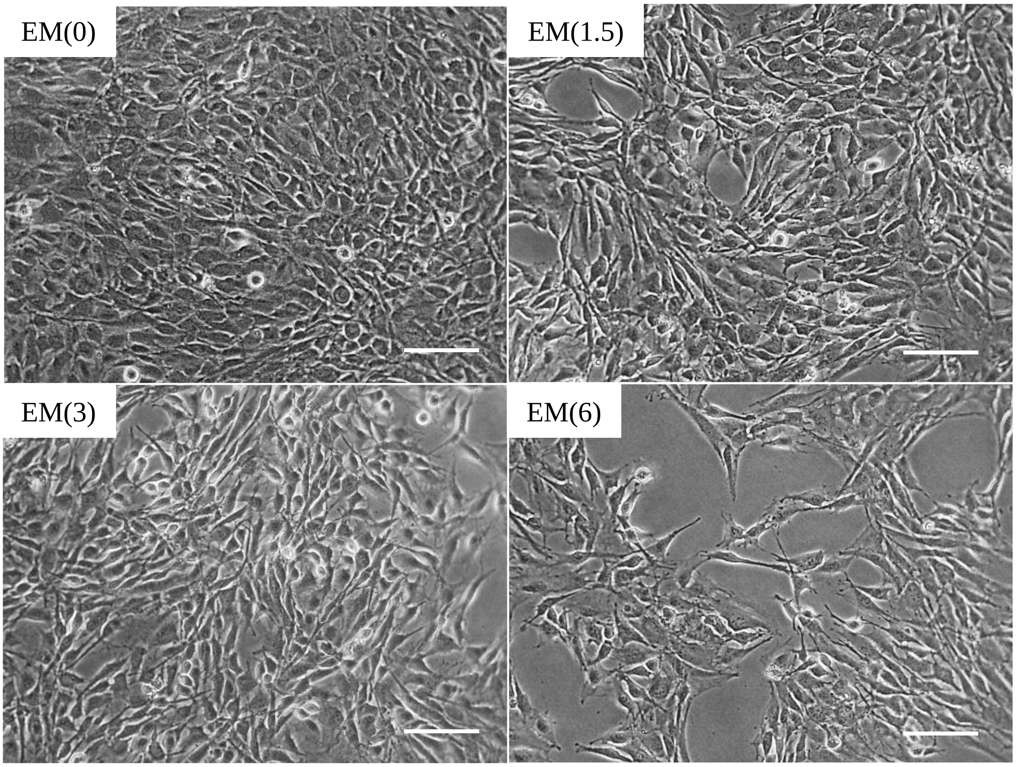

Figure S4. Morphological observation of cells in elution medium of ZOL/β-TCP.
HOS and MC3T3-E1 were cultured in elution medium for 3 days. After culturing, (a) HOS and (b) MC3T3-E1 were observed with phase microscopy. Scale bars; 100 μm.

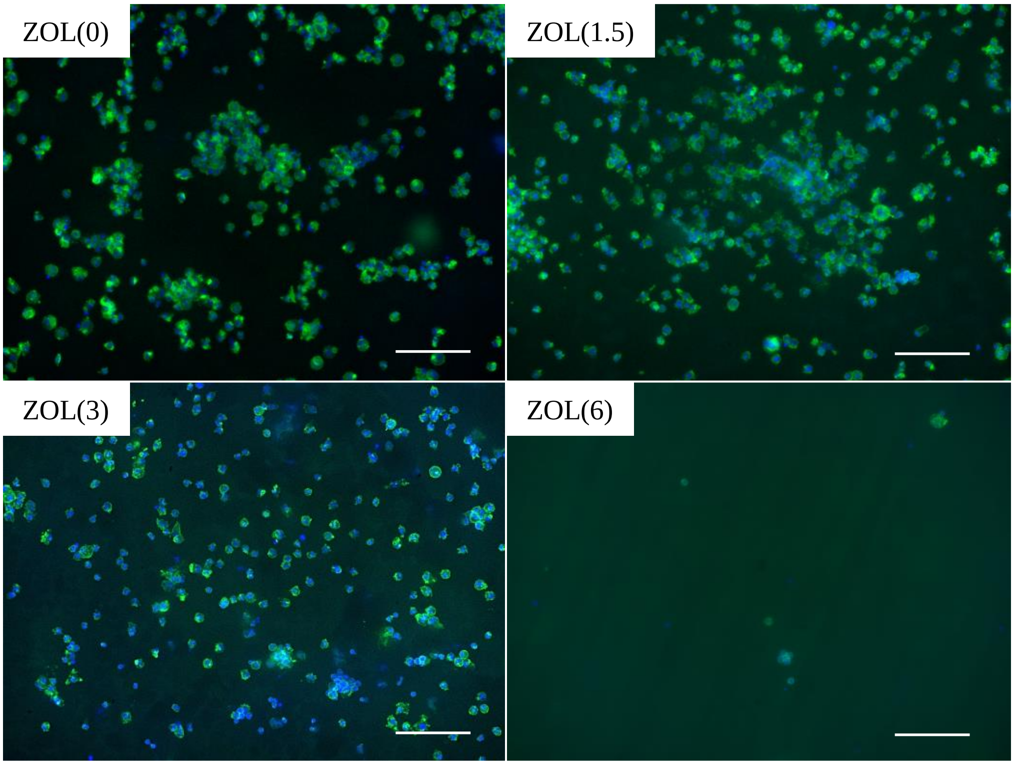

Figure S5. Cells adhesion of mBMSCs on ZOL/β-TCP disc.
mBMSCs were cultured on each ZOL/β-TCP disc for 3 days. After culturing, mBMSCs were stained with Alexa Flour® 488-labeled phalloidin for F-actin (green) and DAPI for nuclei (blue) and observed with fluorescence microscopy. Scale bars; 100 μm

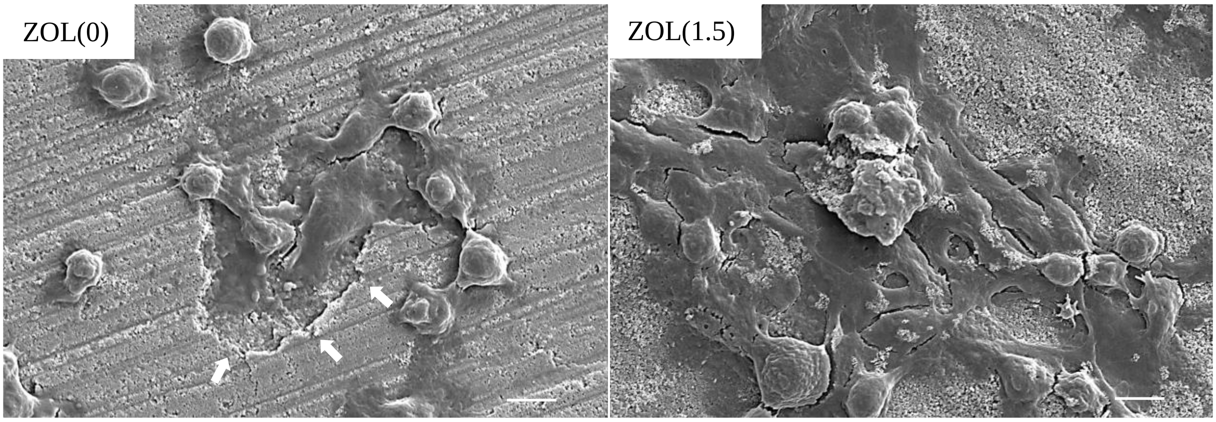

Figure S6. Observation of mBMSCs and pit area.
mBMSCs were cultured on each ZOL/β-TCP disc for 10 days. After culturing, surface of the ZOL/β-TCP disc was observed with SEM and confirmed pit area. White arrows; pit area, scale bars; 10 μm.

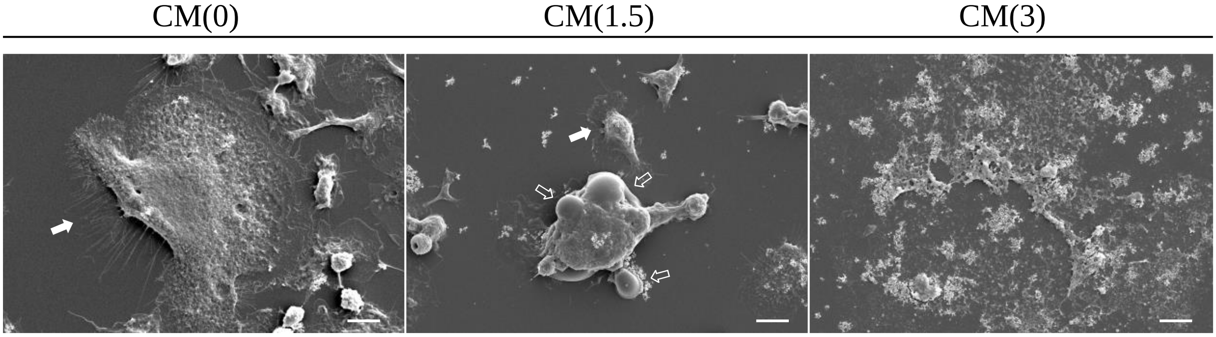

Figure S7. Morphological observation of osteoclasts and apoptotic bodies in osteoclastic conditioned medium. mBMSCs were cultured in osteoclastic (on ZOL/β-TCP disc) conditioned medium for 3 days. mBMSCs were observed with SEM. Scale bars; 10 μm, filled white arrows; osteoclasts, open white arrows; apoptotic bodies.
